# Identification of immune cell function in breast cancer by integrating multiple single-cell data

**DOI:** 10.3389/fimmu.2022.1058239

**Published:** 2022-11-21

**Authors:** Liyuan Zhang, Qiyuan Qin, Chen Xu, Ningyi Zhang, Tianyi Zhao

**Affiliations:** ^1^ Department of Computer Science, Harbin Institute of Technology, Harbin, China; ^2^ Department of Colorectal Surgery, The Sixth Affiliated Hospital, Sun Yat-sen University, Guangzhou, China; ^3^ Center for Bioinformatics, School of Computer Science and Technology, Harbin Institute of Technology, Harbin, China; ^4^ School of Medicine and Health, Harbin Institute of Technology, Harbin, China

**Keywords:** scRNA-seq, integration analysis, immune cells, T cell, functional analysis

## Abstract

Breast cancer has now become the most commonly diagnosed cancer worldwide. It is a highly complex and heterogeneous disease that comprises distinct histological features and treatment response. With the development of molecular biology and immunology, immunotherapy has become a new field of breast cancer treatment. Identifying cell-type-specific genes critical to the immune microenvironment contributes to breast cancer treatment. Single-cell RNA sequencing (scRNA-seq) technology could serve as a powerful tool to analyze cellular genetic information at single-cell resolution and to uncover the gene expression status of each cell, thus allowing comprehensive assessment of intercellular heterogeneity. Because of the influence of sample size and sequencing depth, the specificity of genes in different cell types for breast cancer cannot be fully revealed. Therefore, the present study integrated two public breast cancer scRNA-seq datasets aiming to investigate the functions of different type of immune cells in tumor microenvironment. We identified total five significant differential expressed genes of B cells, T cells and macrophage and explored their functions and immune mechanisms in breast cancer. Finally, we performed functional annotation analyses using the top fifteen differentially expressed genes in each immune cell type to discover the immune-related pathways and gene ontology (GO) terms.

## Introduction

Breast cancer is the leading cause of cancer death among women worldwide. According to cancer statistics, the number of new cases of breast cancer reached 2.26 million in 2020 (1), and breast cancer has now become the most commonly diagnosed cancer worldwide. The most relevant risk factors for breast cancer include the density of breast tissue, estrogen levels, genetic mutations, and lifestyle factors (2). It is a highly complex and heterogeneous disease that comprises distinct histological features, pathologies, and clinical outcomes (3, 4). Due to the vastly different disease manifestations in patients with breast cancer, uniform treatment strategies for patients could fail, resulting in a poor prognosis. With the rapid development of early detection and therapeutic modalities, the survival rates of breast cancer have improved in recent years, but patients still suffer multiple physical and psychological symptoms, which impose a huge burden on families and society (5).

Currently, breast cancer is classified into different molecular subtypes based on molecular markers for estrogen receptor (ER), progesterone receptor (PR), and human epidermal growth factor receptor 2 (HER2) (6). In clinical practice, different breast cancer subtypes have distinct phenotypes, risk profiles, and prognoses. Triple-negative breast cancer (TNBC) is a specific subtype of breast cancer characterized as HER2-negative, PR-negative, and ER-negative (7). Compared to other subtypes of breast cancer, TNBC tends to grow and metastasize faster, resulting in a worse prognosis (8). Given that there are different subtypes of breast cancer, rationalized therapy is required to be conducted according to the characterization of the disease and the clinical behavior of individual patients (9). However, a major challenge in implementing precision therapies is our lack of insights into breast cancer ecosystems. Thus, it is essential to conduct a comprehensive analysis of cellular compositions to reveal the microenvironmental characteristics of breast cancer.

During the progression of breast cancer, breast tumors show multiple genetic changes in spatial and temporal dimensions, such as clonal evolution and mutational diversification, leading to genotypic and phenotypic heterogeneities (10). Single-cell RNA sequencing technology could serve as a powerful tool to analyze cellular genetic information at single-cell resolution and to uncover the gene expression status, thus allowing a comprehensive assessment of tumor heterogeneity (11). Furthermore, it can also monitor rare cellular mutations during tumorigenesis, such as the acquisition of invasive and metastatic capabilities, as well as the infiltration of immune cells (12). Though breast cancer has been generally considered non-immunogenic for a long time, the accumulation of evidence has indicated that immune components in the tumor microenvironment played a vital role in various aspects of breast cancer (13–15). For instance, the degree of infiltration of follicular helper CD4 T cells can be used as an indicator to reflect and predict overall survival for HER2-positive breast cancer and TNBC (16). Tumor-educated B cells enable the production of IgG to selectively promote the metastasis of breast cancer in lymph nodes (17). Therefore, investigating the functions of different types of cells in the immune ecosystem can add more dimensions to the understanding of breast cancer.

With the rapid rise of high-throughput sequencing technology, numerous studies based on single-cell RNA sequencing (scRNA-seq) have emerged for revealing the functions and mechanisms of immune cells in breast cancer. Savas et al. (18) found that CD8+ T cells were characterized by tissue-resident memory T-cell differentiation, which contributed to high levels of immune checkpoint molecules by performing scRNA-seq of 6,311 T cells from patients with breast cancer. Hu et al. (19) revealed that tumor-infiltrated B cells were more similar to mature and memory B cells and that subgroups of B cells in the microenvironment of breast cancer were related to immunosurveillance. Additionally, other types of immune cells such as neutrophils and macrophages have the ability to influence biological processes associated with breast cancer by inducing the expression of transcription factors.

In the present study, we integrated and analyzed the single-cell RNA sequencing datasets through bioinformatics methods to explore the tumor immune microenvironment of breast cancer. We focused on the composition of the immune cells in breast cancer and the functions of cell type-specific genes. Furthermore, we applied functional enrichment to discover the pathways in all types of immune cells. Overall, our study may add a new dimension for further research on the immune ecosystem and immunotherapy for breast cancer.

## Material and methods

### Dataset

Two public datasets of single-cell RNA sequencing data were downloaded from the Gene Expression Omnibus (GEO) database (GSE75688 and GSE118389) (20, 21). For the GSE75688 dataset, Chung et al. (20) obtained 515 cells from 11 patients with four subtypes of breast cancer: HER2, luminal A, luminal B, and TNBC. The single-cell RNA sequencing data were collected from two metastatic lymph nodes (BC03LN and BC07LN) and 11 primary tumor specimens (BC01–BC11). For the GSE118389 dataset, Karaayvaz et al. (21) collected 1,534 cells in six fresh tumors (PT039, PT058, PT081, PT084, PT089, and PT0126) from patients with primary, non-metastatic triple-negative breast cancer.

### Quality control

We already performed quality control on the expression matrix in the public GSE118389 dataset, and we only considered the GSE75688 dataset. For the GSE75688 dataset, we implemented quality control to filter out the low-quality cells. We first detected the gene expression of each cell and excluded cells or cell doublets with the following criteria: cells with few genes per cell or many molecules per cell and cells that had >5% mitochondrial counts or >10% ribosomal counts. Then, we normalized and log-transformed the filtered gene expression matrix. In addition, we also log-transformed the expression data of the GSE118389 dataset after the filtration.

### Dimension reduction, cell clustering and identification

First, the FindVariableFeatures function was applied to discover a subset of features that exhibit high variation in the dataset. A total of 3,000 variably expressed genes were returned, and these features will be used in the dimensionality reduction process. Then, a linear transformation was performed to scale the expression of each gene. The ScaleData was used to shift and scale the expression data so that the mean expression across cells is 0, and the variance across cells is 1. Next, principal component analysis (PCA) was performed on the scaled data with the previously determined variable features as input. PCA is a linear dimensionality reduction method, and the top principal components could serve as important metafeatures to essentially represent the compression of the dataset (22). After the implementation of dimensionality reduction, the data integration on two datasets of breast cancer was presented. The Seurat objects of each dataset were first merged in order to obtain a list of Seurat objects. This list of Seurat objects was taken as input to identify and score anchors by using the FindIntegrationAnchors function. Anchors are cross-dataset pairs of cells that are in a matched biological state to correct batch effect between datasets (referred to as “reference” and “query” datasets). Their batch vectors could be calculated, and then these vectors were used to correct the gene expression values of the subset of cells being anchored. Then, the Seurat function IntegrateData was applied to integrate the two datasets together based on the anchors. For the integrated data after batch effect correction, the top principal components of the PCA were taken as input for the function FindNeighbors, and the function FindClusters was used to cluster cells together. To intuitively observe the cell clustering, uniform manifold approximation and projection (UMAP) and t-distributed stochastic neighbor embedding (t-SNE) were applied to present different cell clusters in a two-dimensional panel (22, 23). For the purpose of annotation for canonical cell clusters, the FindAllMarkers function was used to identify the marker genes of each cellular cluster.

### Differentially expressed analysis of immune cells

To identify cluster-specific differentially expressed genes for immune cells, the FindMarkers function was applied to calculate the value of the log2 fold change. The significance of the difference was determined by using the Wilcoxon rank-sum test with the Bonferroni correction.

### Pathway and functional annotation analyses

**Figure 1:**
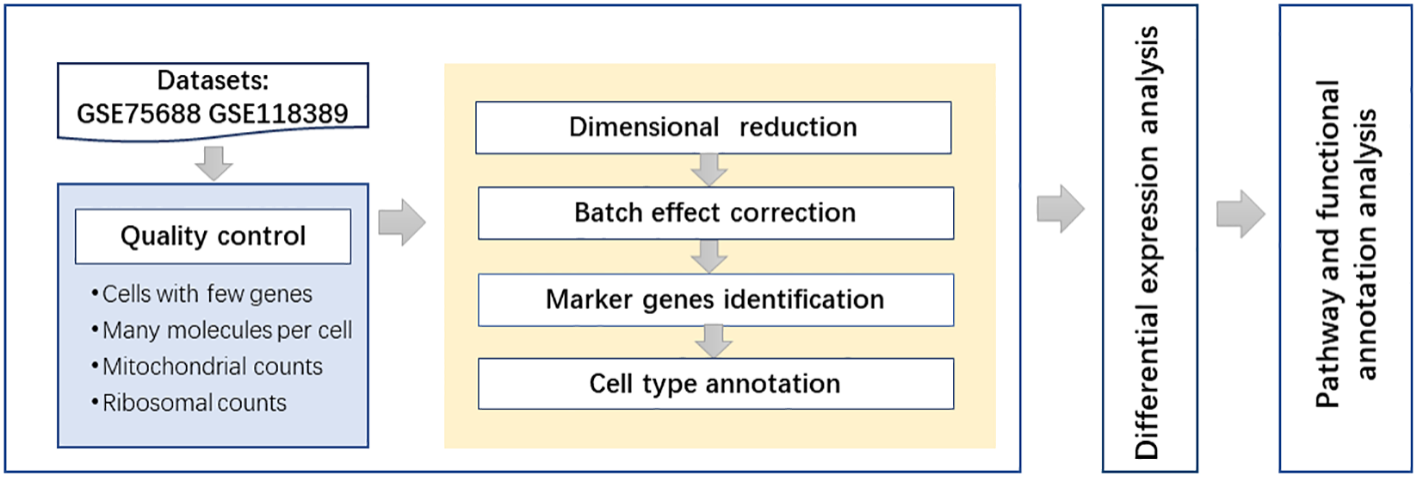
The flowchart of the overall analysis.

Gene Ontology (GO) (24) terms and Kyoto Encyclopedia of Genes and Genomes (KEGG) (25) pathways were discussed in this study. GO terms were used for describing the functions of the genes and their product properties. The Gene Ontology knowledgebase provides three aspects of functional annotation: biological process, cellular component, and molecular function classifications. KEGG is a widely used database resource that stores information on high-level functions for systematic analysis of genes. The DAVID (https://david.ncifcrf.gov/) functional annotation tool was used to explore the GO terms and KEGG pathways related to differentially expressed genes in specific cell types. Pathways and GO terms were considered to have significance at the level of corrected p-value <0.05. The flowchart of this study is shown in **Figure 1**.

## Results

### Processing of single-cell RNA sequencing data

**Figure 2:**
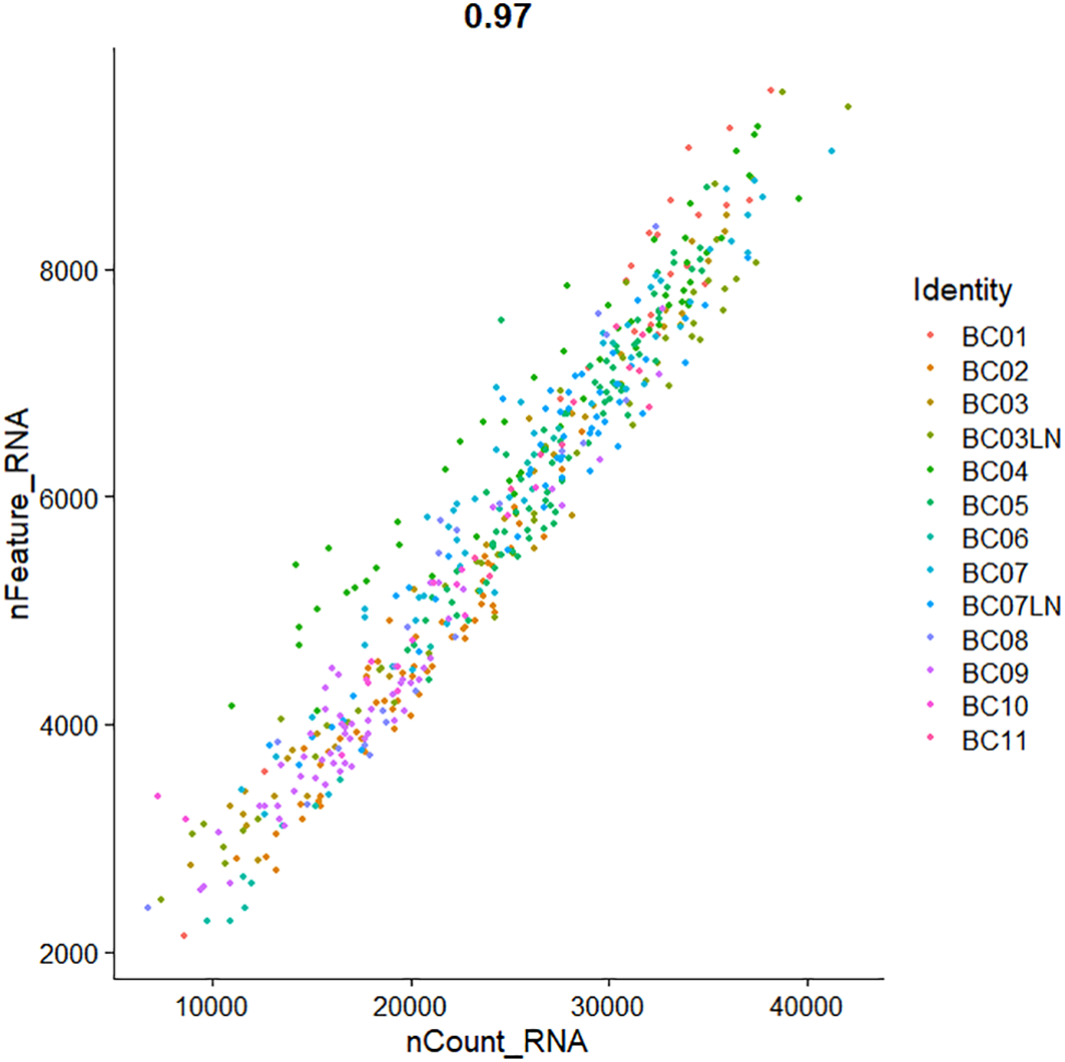
The Scatter plot of the feature–feature relationships in GSE75688 dataset after quality control.

Quality control of single-cell transcriptome data from four breast cancer patients from the GSE75688 dataset was performed in this study. For the matrix data, the threshold for quality control was measured by observing the amount of gene expression. The total count of genes measured in the data was focused on, the total count of transcript molecules was measured, and the proportion of reads was localized to mitochondrial and ribosomal genomic genes. It was observed that the percentage of mitochondrial gene reads was <5%, which was within the normal range, so they were not processed. In addition, there were data with the ribosomal gene reads percentage above 10%, which needed to be filtered out. The total number of genes measured in the cells and the total number of transcript molecules measured had several smaller values, which could be seen that the cells express fewer genes and that there may be invalid reverse transcription (low-quality cells or empty droplets) during sequencing. In order to filter out the invalid data, the quality control parameters were set as nFeature_RNA values >2,800 and nCount_RNA values >9,000. As shown in **Figure 2**, the FeatureScatter function in the Seurat package was used to visualize feature–feature relationships in the GSE75688 dataset. For the expression data of the GSE75688 dataset after the filtration, the normalization was completed by the NormalizeData function. In addition, the expression data of the GSE75688 and GSE118389 datasets were also log-transformed. Log-transformation could attenuate mean–variance relationships in single-cell transcriptome sequencing data and reduce the dispersion of gene expression.

### Principal component analysis and batch effect correction

**Figure 3:**
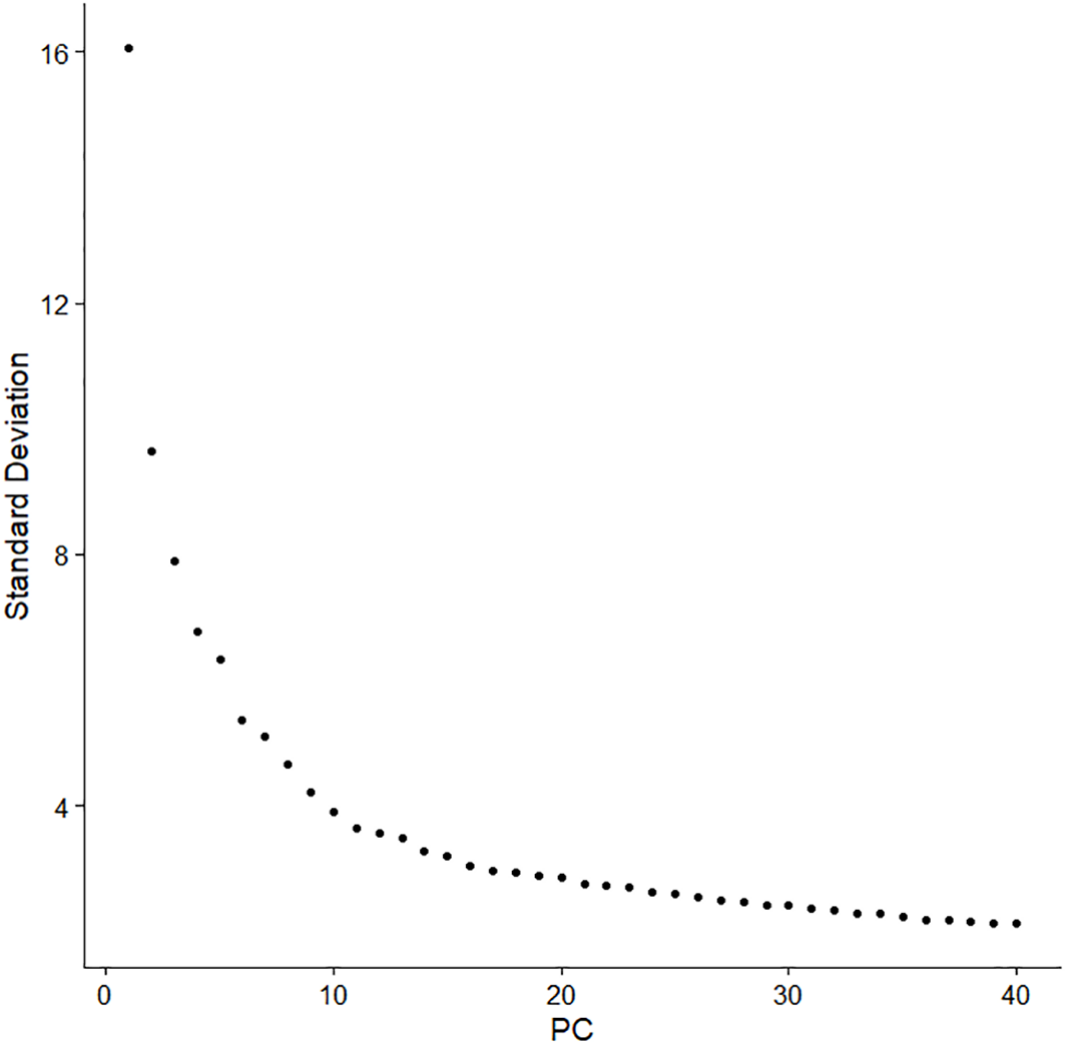
The elbow plot of the principal component analysis.

**Figure 4:**
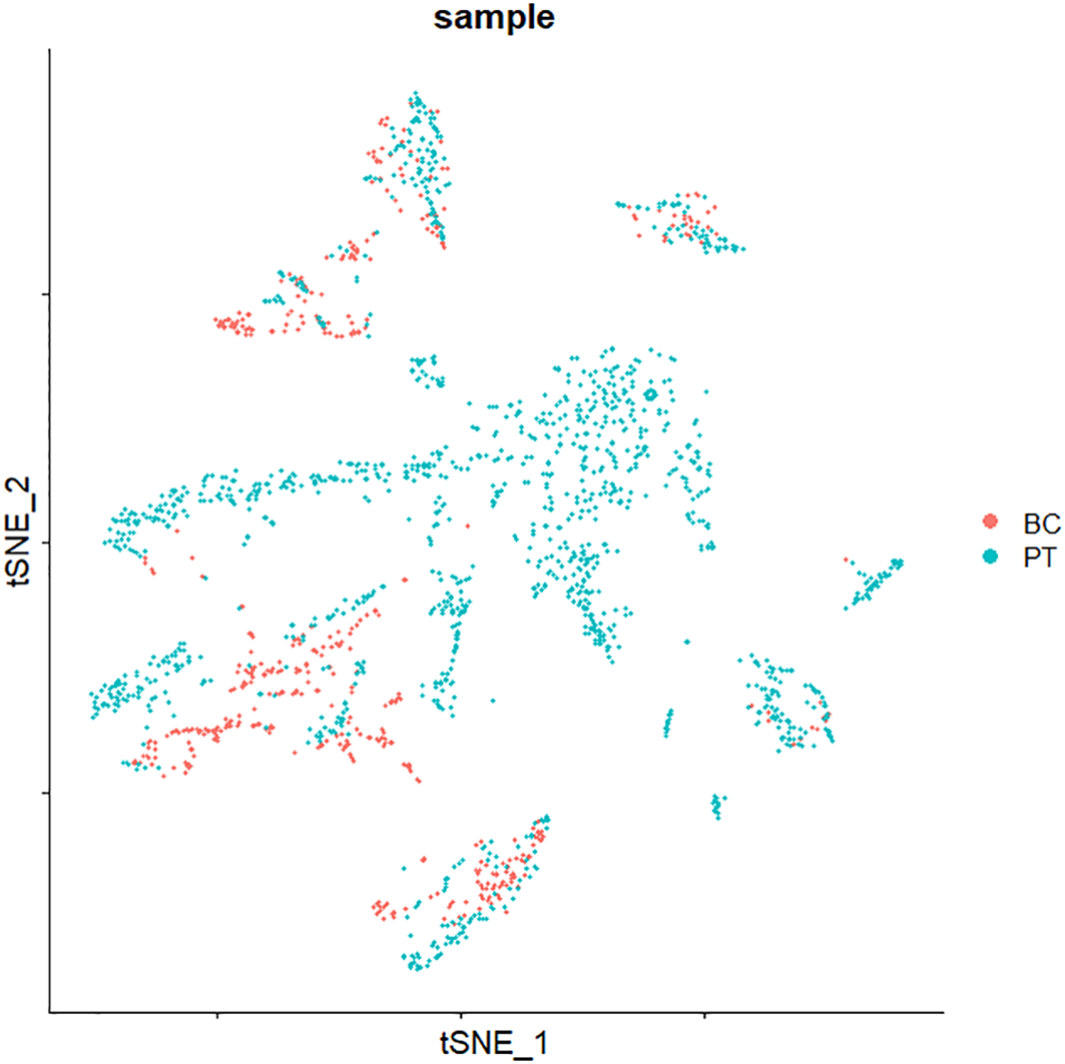
t-SNE of the integration for datasets GSE75688 and GSE118389. t-SNE, t-distributed stochastic neighbor embedding.

Subsequently, principal component analysis was used to perform linear dimensionality reduction, and a set of linearly independent variables, i.e., principal component (PC), was constructed by orthogonal transformation, so as to retain most of the feature dimensions and ignore the less influential ones. In this study, the Elbowplot function was used to determine the number of principal components (**Figure 3**). The elbow plot showed the contribution of each principal component, which was ranked according to the percentage of variance. It can be seen that an “inflection point” around PC10 indicates that most of the true signal was captured in the first dozen principal components, and 13 principal components were finally selected for subsequent analysis. The PCA was performed on the expression data for dimensionality reduction analysis. Furthermore, the phenomenon of batch effect existed in these two sets of cross-platform scRNA-seq data, and the correction of batch effect was essential before data integration. The result of the integration after batch effect correction is shown in **Figure 4**.

### Cell clustering and identification

**Figure 5:**
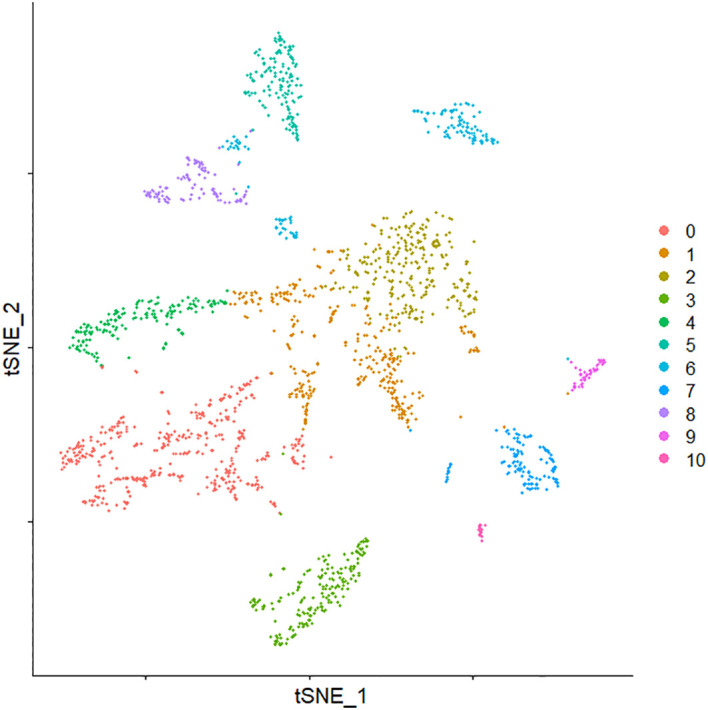
t-SNE of cell clustering result. t-SNE, t-distributed stochastic neighbor embedding.

The clustering of cells is the basis for cell type identification and is a key step in single-cell RNA sequencing to reveal heterogeneity. A graph-based clustering algorithm was used to cluster breast tissue cells so that similar cells were clustered together. The k-nearest neighbors of each cell were found in the feature space after principal component analysis, and their edge weights were refined for the shared overlap of any two cells in the neighborhood to divide the cells into highly interconnected communities, and then the clustering of cells was completed using the Louvain community discovery algorithm based on modularity. In our experiments, the resolution parameter was set to 0.4, and all cells were divided into 11 cell clusters. The clustered cells were visualized in a two-dimensional panel by using non-linear dimensionality reduction t-SNE, as shown in **Figure 5**. Subsequently, the marker genes for each cell type were identified, and cell type identity was assigned to clusters by comparing marker gene sets with other databases.

### Differential expression analysis and enrichment analysis

**Figure 6:**
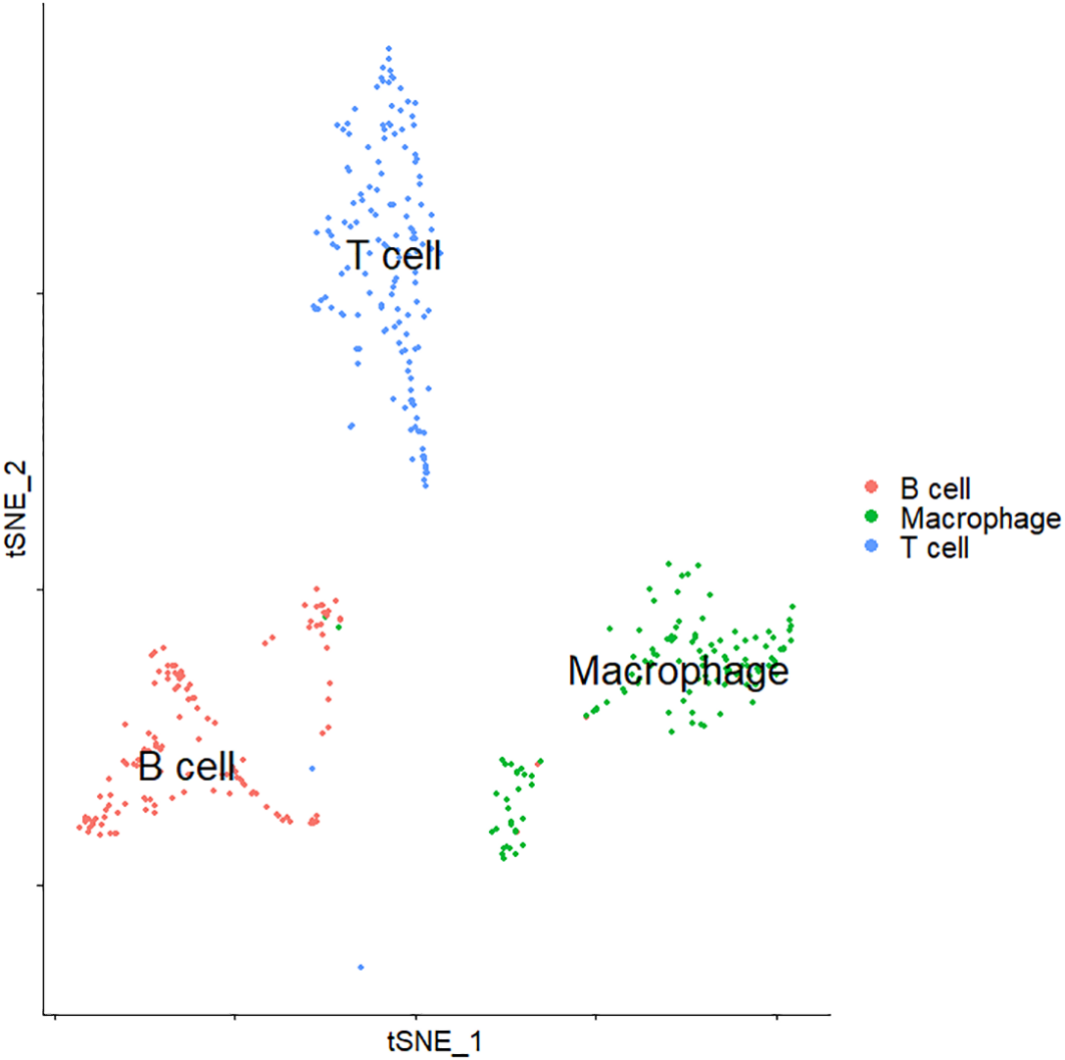
t-SNE visualization of the cluster of immune cells. t-SNE, t-distributed stochastic neighbor embedding.

We focused on the differential expression analysis of immune cell populations by first extracting immune cells in all cell types (**Figure 6**). Immune cells include three types of cells: B cells, T cells, and macrophages. We compared log-transformed and normalized expression values of genes that were expressed in at least 25% of cells between the three types of immune cell clusters to identify differentially expressed genes in each cell type. TNFRSF13B was the most specifically expressed gene in cluster B cells. For T cells, UBASH3A, TRAT1, and GZMA could serve as the significantly differentially expressed genes. Additionally, we identified CD14 as the specifically expressed gene for macrophages. The expression levels of these cell type-specific genes may influence the functional regulation of different types of immune cells in the development and progression of breast cancer.

**Figure 7:**
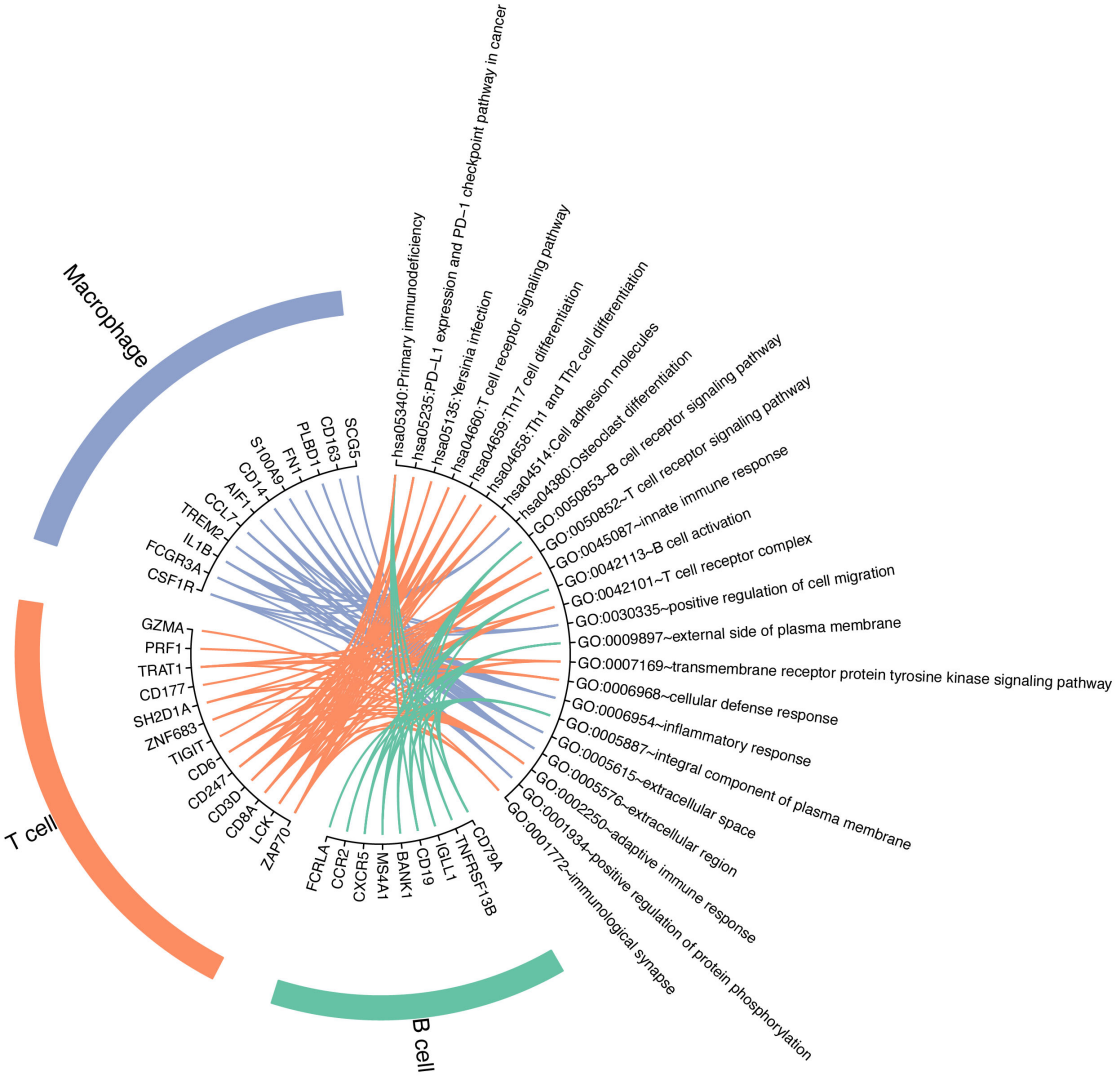
GO and KEGG enrichment analysis of differentially expressed genes in immune cell types.

Next, we performed functional enrichment analysis using the top 15 differentially expressed genes for each immune cell type (**Supplementary Material**). The pathways and GO terms enriched by differentially expressed genes of each immune cell type are shown in **Figure 7**. For the annotation for KEGG pathway analysis, differentially expressed genes in T cells were enriched in more pathways compared with B cells and macrophages. The significantly differentially expressed gene TNFRSF13B in B cells was enriched in the primary immunodeficiency pathway. Differentially expressed genes CSF1R, FCGR3A, IL1B, and TREM2 of macrophages were enriched only in the osteoclast differentiation pathway. For T cells, genes were preferentially enriched on the T-cell receptor signaling pathway and primary immunodeficiency. In addition, type-specific genes of T cells are also enriched in other types of pathways, such as PD-L1 expression and PD-1 checkpoint pathway in cancer and Th1 and Th2 cell differentiation. It should be noted that Th1 or Th2 effector subsets are differentiated from CD4 helper T (Th) cells after activation. GO functional enrichment analysis results showed that the differentially expressed genes (DEGs) in T cells were mainly correlated with immune response and T-cell receptor, including adaptive immune response and innate immune response, T-cell receptor signaling pathway, and T-cell receptor complex. In addition, the GO terms of B cells were mostly related to the plasma membrane and the activities of B cells. The DEGs in B cells can play a role in the B-cell receptor signaling pathway and B-cell activation. The inflammatory response was mainly associated with macrophages.

## Discussion

The immune microenvironment of cancer is relevant in terms of cancer progression, immune escape, and treatment resistance. Due to the high heterogeneity of immune cells in the tumor microenvironment, single-cell RNA sequencing technology has greatly facilitated the study of cancer evolutionary processes and cell types associated with the cancer microenvironment while enabling the discovery of type-specific genes of different cell types at an extremely fine scale, bringing new insights on cancer diagnosis and treatment (26). In our study, we integrated and analyzed two scRNA-seq datasets of breast cancer to characterize the gene expression of immune cell types in the immune microenvironment of breast cancer tissues and discover the pathways and potential functions associated with type-specific genes.

ScRNA-seq can serve as a useful tool to identify variations in gene expression in immune cell populations, which might contribute to discovering the mechanisms of complex immunological responses. In order to explore the key genes for the prognosis and treatment of breast cancer, the gene expression of cell types in the immune microenvironment of breast cancer tissues was characterized, and the pathways and potential functions associated with type-specific genes were discovered (27). The result of the single-cell RNA-seq analysis has shown that three types of immune cells in breast cancer were identified, including B cells, T cells, and macrophages. We analyzed the significant marker genes of each cell type by performing differential expression analysis. TNFRSF13B is the significant marker gene in B cells for breast cancer. TNFRSF13B is a member of the TNF receptor superfamily existing in peripheral B-cell homeostasis, which could mediate calcineurin-dependent activation of NF-AT and AP-1 to stimulate B cells (28). In T cells, NF-AT proteins are activated following T-cell receptor ligation, and the proteins have the ability to regulate the activation, differentiation, and development of T cells (29). Additionally, it has been confirmed that silencing of the cytokine receptor TNFRSF13B might serve as a new therapeutic target for triple-negative breast cancer, as it could result in significant death in breast cancer cell lines. Meanwhile, we identified the top three differentially expressed genes (UBASH3A, TRAT1, and GZMA) in T cells. UBASH3A is a family member of the T-cell ubiquitin ligand (TULA) family, which facilitates growth factor withdrawal-induced apoptosis in T cells. It is obvious that the apoptosis of immune cells enables tumor cells to spread and metastasize more rapidly. TRAT1 is the T-cell receptor-associated transmembrane adaptor, and it has been confirmed to be upregulated in T cells of breast cancer in the previous study (20). TRAT1 gene was found to be differentially expressed in the lymph nodes in patients with metastatic breast cancer. Additionally, higher levels of GZMA may represent higher levels of cytotoxic T-cell infiltration, leading to better survival rates of breast cancer (30). For macrophages, the level of infiltration of CD14-positive macrophages can serve as an indicator of early recurrence of breast cancer (31). These results are in the initial analysis and still need further experimental verification. However, these results expand our understanding of the functions and regulation mechanism of these DEGs in different immune cell types.

Many signaling pathways are involved in the development and evolution of cancer. Based on the top 15 differentially expressed genes in each immune cell type, we further explored the GO terms and KEGG pathways related to the specific cell-type genes. We focused on the enrichment analysis of DEGs in T cells. A total of seven KEGG pathways had been enriched, including the T-cell receptor signaling pathway, primary immunodeficiency, PD-L1 expression and PD-1 checkpoint pathway in cancer, Th1 and Th2 cell differentiation, Th17 cell differentiation, *Yersinia* infection, and cell adhesion molecules. Some specific T-cell receptor signaling pathways might have an impact on the progression and development of breast cancer. For instance, a discrete mode of T-cell receptor (TCR) signaling can regulate Treg cell differentiation to affect metabolism, cell adhesion, and cell migration (32). A strong Th1 response is essential for effective antitumor immunity, and higher levels of Th1 cytokines are associated with ER-negative and triple-negative breast cancers (33). For B cells, the DEGs were enriched on the primary immunodeficiency pathway. The common variable immunodeficiency may lead to breast cancer (34). In addition, genes CSF1R, FCGR3A, IL1B, and TREM2 of macrophages are enriched only in the osteoclast differentiation pathway. Osteoclast displays a wide heterogeneity and plasticity and is involved in phagocytosis and innate immune responses (35). Osteoclasts and breast cancer cells can collaborate with each other, which results in the formation of breast cancer osteolytic bone metastasis (36). The GO terms of T cells were mainly relevant to immunization, such as adaptive immune response, innate immune response, and the immunological synapse. T cells of the innate immune system are involved in the response to the threat of breast tumor cells. Moreover, infiltration of specific T-follicular helper cells in human breast cancer may promote effective adaptive immunity (37). DEGs in B cells are preferentially enriched in the B-cell receptor signaling pathway in GO enrichment analysis, which is highly relevant to B-cell development and adaptive immunity in cancer (38). Furthermore, GO terms for macrophages are mainly related to the inflammatory response. Some signaling molecule triggers such as Biglycan can regulate inflammation through CD14 to influence tumor progression (39). Thus, functional analysis of differentially expressed genes for immune cell types can add a new dimension to the diagnosis and prognosis of breast cancer. It also provides a theoretical basis for the potential clinical translation of breast cancer.

In conclusion, we uncovered and dissected significantly differentially expressed genes in different immune cell types and their function annotation by integrating and analyzing single-cell transcriptome sequencing data of breast cancer. However, more research is still required to investigate the genes and their potential functions in the breast tumor immune microenvironment for further elucidating the key messages behind the subtype-specific differences of immune cells.

## Data availability statement

The data can be accessed by:https://www.ncbi.nlm.nih.gov/geo/query/acc.cgi?acc=GSE75688, https://www.ncbi.nlm.nih.gov/geo/query/acc.cgi?acc=GSE118389.

## Author contributions

LZ analyzed the data, and QQ wrote the article. CX collected the datasets, and NZ revised the pipeline. TZ designed the whole work. All authors contributed to the article and approved the submitted version.

## Funding

This work has been supported by the National Key Research and Development Program of China (No: 2021YFF1200105), the Natural Science Foundation of China (No: 62102116) and Interdisciplinary Research Foundation of HIT.

## Conflict of interest

The authors declare that the research was conducted in the absence of any commercial or financial relationships that could be construed as a potential conflict of interest.

## Publisher’s note

All claims expressed in this article are solely those of the authors and do not necessarily represent those of their affiliated organizations, or those of the publisher, the editors and the reviewers. Any product that may be evaluated in this article, or claim that may be made by its manufacturer, is not guaranteed or endorsed by the publisher.

## References

[1] Ferlay J Colombet M Soerjomataram I Parkin DM Pineros M Znaor A . Cancer statistics for the year 2020: An overview. Int J Cancer (2021) 149(4):778–89. doi: 10.1002/ijc.33588 33818764

[2] Cardoso F Kyriakides S Ohno S Penault-Llorca F Poortmans P Rubio IT . Early breast cancer: Esmo clinical practice guidelines for diagnosis, treatment and follow-updagger. Ann Oncol (2019) 30(8):1194–220. doi: 10.1093/annonc/mdz173 31161190

[3] Martelotto LG Ng CK Piscuoglio S Weigelt B Reis-Filho JS . Breast cancer intra-tumor heterogeneity. Breast Cancer Res (2014) 16(3):210. doi: 10.1186/bcr3658 25928070 PMC4053234

[4] Zardavas D Irrthum A Swanton C Piccart M . Clinical management of breast cancer heterogeneity. Nat Rev Clin Oncol (2015) 12(7):381–94. doi: 10.1038/nrclinonc.2015.73 25895611

[5] Khan JS Ladha KS Abdallah F Clarke H . Treating persistent pain after breast cancer surgery. Drugs (2020) 80(1):23–31. doi: 10.1007/s40265-019-01227-5 31784873

[6] Weigelt B Reis-Filho JS . Molecular profiling currently offers no more than tumour morphology and basic immunohistochemistry. Breast Cancer Res (2010) 12 Suppl 4:S5. doi: 10.1186/bcr2734 21172089 PMC3005725

[7] Yin L Duan JJ Bian XW Yu SC . Triple-negative breast cancer molecular subtyping and treatment progress. Breast Cancer Res (2020) 22(1):61. doi: 10.1186/s13058-020-01296-5 32517735 PMC7285581

[8] De Laurentiis M Cianniello D Caputo R Stanzione B Arpino G Cinieri S . Treatment of triple negative breast cancer (Tnbc): Current options and future perspectives. Cancer Treat Rev (2010) 36 Suppl 3:S80–6. doi: 10.1016/S0305-7372(10)70025-6 21129616

[9] Akram M Iqbal M Daniyal M Khan AU . Awareness and current knowledge of breast cancer. Biol Res (2017) 50(1):33. doi: 10.1186/s40659-017-0140-9 28969709 PMC5625777

[10] Polyak K . Breast cancer: Origins and evolution. J Clin Invest (2007) 117(11):3155–63. doi: 10.1172/JCI33295 PMC2045618 17975657

[11] Zhang X Marjani SL Hu Z Weissman SM Pan X Wu S . Single-cell sequencing for precise cancer research: Progress and prospects. Cancer Res (2016) 76(6):1305–12. doi: 10.1158/0008-5472.CAN-15-1907 26941284

[12] Kuipers J Jahn K Beerenwinkel N . Advances in understanding tumour evolution through single-cell sequencing. Biochim Biophys Acta Rev Cancer (2017) 1867(2):127–38. doi: 10.1016/j.bbcan.2017.02.001 PMC5813714 28193548

[13] Savas P Salgado R Denkert C Sotiriou C Darcy PK Smyth MJ . Clinical relevance of host immunity in breast cancer: From tils to the clinic. Nat Rev Clin Oncol (2016) 13(4):228–41. doi: 10.1038/nrclinonc.2015.215 26667975

[14] Loi S Sirtaine N Piette F Salgado R Viale G Van Eenoo F . Prognostic and predictive value of tumor-infiltrating lymphocytes in a phase iii randomized adjuvant breast cancer trial in node-positive breast cancer comparing the addition of docetaxel to doxorubicin with doxorubicin-based chemotherapy: Big 02-98. J Clin Oncol (2013) 31(7):860–7. doi: 10.1200/JCO.2011.41.0902 23341518

[15] Cimino-Mathews A Foote JB Emens LA . Immune targeting in breast cancer. Oncol (Williston Park) (2015) 29(5):375–85. 25979549

[16] Vonderheide RH Domchek SM Clark AS . Immunotherapy for breast cancer: What are we missing? Clin Cancer Res (2017) 23(11):2640–6. doi: 10.1158/1078-0432.CCR-16-2569 PMC5480967 28572258

[17] Gu Y Liu Y Fu L Zhai L Zhu J Han Y . Tumor-educated b cells selectively promote breast cancer lymph node metastasis by Hspa4-targeting igg. Nat Med (2019) 25(2):312–22. doi: 10.1038/s41591-018-0309-y 30643287

[18] Savas P Virassamy B Ye C Salim A Mintoff CP Caramia F . Single-cell profiling of breast cancer T cells reveals a tissue-resident memory subset associated with improved prognosis. Nat Med (2018) 24(7):986–93. doi: 10.1038/s41591-018-0078-7 29942092

[19] Hu Q Hong Y Qi P Lu G Mai X Xu S . Atlas of breast cancer infiltrated b-lymphocytes revealed by paired single-cell rna-sequencing and antigen receptor profiling. Nat Commun (2021) 12(1):2186. doi: 10.1038/s41467-021-22300-2 33846305 PMC8042001

[20] Chung W Eum HH Lee HO Lee KM Lee HB Kim KT . Single-cell rna-seq enables comprehensive tumour and immune cell profiling in primary breast cancer. Nat Commun (2017) 8:15081. doi: 10.1038/ncomms15081 28474673 PMC5424158

[21] Karaayvaz M Cristea S Gillespie SM Patel AP Mylvaganam R Luo CC . Unravelling subclonal heterogeneity and aggressive disease states in tnbc through single-cell rna-seq. Nat Commun (2018) 9(1):3588. doi: 10.1038/s41467-018-06052-0 30181541 PMC6123496

[22] Kobak D Berens P . The art of using T-sne for single-cell transcriptomics. Nat Commun (2019) 10(1):5416. doi: 10.1038/s41467-019-13056-x 31780648 PMC6882829

[23] Becht E McInnes L Healy J Dutertre CA Kwok IWH Ng LG . Dimensionality reduction for visualizing single-cell data using umap. Nat Biotechnol (2018) 37:38–44. doi: 10.1038/nbt.4314 30531897

[24] Ashburner M Ball CA Blake JA Botstein D Butler H Cherry JM . Gene ontology: Tool for the unification of biology. Gene Ontol Consortium. Nat Genet (2000) 25(1):25–9. doi: 10.1038/75556 PMC3037419 10802651

[25] Kanehisa M Goto S . Kegg: Kyoto encyclopedia of genes and genomes. Nucleic Acids Res (2000) 28(1):27–30. doi: 10.1093/nar/28.1.27 10592173 PMC102409

[26] Cheng N Chen C Li C Huang J . Inferring cell-Type-Specific genes of lung cancer based on deep learning. Curr Gene Ther (2022) 22(5):439–48. doi: 10.2174/1566523222666220324110914 35331109

[27] Zhao T Liu J Zeng X Wang W Li S Zang T . Prediction and collection of protein-metabolite interactions. Brief Bioinform (2021) 22(5):bbab014. doi: 10.1093/bib/bbab014 33554247

[28] Bogaert DJ Dullaers M Lambrecht BN Vermaelen KY De Baere E Haerynck F . Genes associated with common variable immunodeficiency: One diagnosis to rule them all? J Med Genet (2016) 53(9):575–90. doi: 10.1136/jmedgenet-2015-103690 27250108

[29] Macian F . Nfat proteins: Key regulators of T-cell development and function. Nat Rev Immunol (2005) 5(6):472–84. doi: 10.1038/nri1632 15928679

[30] Fisler DA Sikaria D Yavorski JM Tu YN Blanck G . Elucidating feed-forward apoptosis signatures in breast cancer datasets: Higher fos expression associated with a better outcome. Oncol Lett (2018) 16(2):2757–63. doi: 10.3892/ol.2018.8957 PMC6036554 30013671

[31] Heiskala M Leidenius M Joensuu K Heikkila P . High expression of Ccl2 in tumor cells and abundant infiltration with Cd14 positive macrophages predict early relapse in breast cancer. Virchows Arch (2019) 474(1):3–12. doi: 10.1007/s00428-018-2461-7 30368555

[32] Li MO Rudensky AY . T Cell receptor signalling in the control of regulatory T cell differentiation and function. Nat Rev Immunol (2016) 16(4):220–33. doi: 10.1038/nri.2016.26 PMC4968889 27026074

[33] Hong CC Yao S McCann SE Dolnick RY Wallace PK Gong Z . Pretreatment levels of circulating Th1 and Th2 cytokines, and their ratios, are associated with er-negative and triple negative breast cancers. Breast Cancer Res Treat (2013) 139(2):477–88. doi: 10.1007/s10549-013-2549-3 PMC3912696 23624818

[34] Shapiro RS . Malignancies in the setting of primary immunodeficiency: Implications for Hematologists/Oncologists. Am J Hematol (2011) 86(1):48–55. doi: 10.1002/ajh.21903 21120868

[35] Madel MB Ibanez L Wakkach A de Vries TJ Teti A Apparailly F . Immune function and diversity of osteoclasts in normal and pathological conditions. Front Immunol (2019) 10:1408. doi: 10.3389/fimmu.2019.01408 31275328 PMC6594198

[36] Le Pape F Vargas G Clezardin P . The role of osteoclasts in breast cancer bone metastasis. J Bone Oncol (2016) 5(3):93–5. doi: 10.1016/j.jbo.2016.02.008 PMC5063222 27761364

[37] Noel G Fontsa ML Garaud S De Silva P de Wind A Van den Eynden GG . Functional Th1-oriented T follicular helper cells that infiltrate human breast cancer promote effective adaptive immunity. J Clin Invest (2021) 131(19):e139905. doi: 10.1172/JCI139905 34411002 PMC8483751

[38] Burger JA Wiestner A . Targeting b cell receptor signalling in cancer: Preclinical and clinical advances. Nat Rev Cancer (2018) 18(3):148–67. doi: 10.1038/nrc.2017.121 29348577

[39] Roedig H Damiescu R Zeng-Brouwers J Kutija I Trebicka J Wygrecka M . Danger matrix molecules orchestrate Cd14/Cd44 signaling in cancer development. Semin Cancer Biol (2020) 62:31–47. doi: 10.1016/j.semcancer.2019.07.026 31412297

